# Kinetic Modelling the Solid–Liquid Extraction Process of Scandium from Red Mud: Influence of Acid Composition, Contact Time and Temperature

**DOI:** 10.3390/ma16216998

**Published:** 2023-11-01

**Authors:** Diana Daminescu, Narcis Duteanu, Mihaela Ciopec, Adina Negrea, Petru Negrea, Nicoleta Sorina Nemeş, Bogdan Pascu, Radu Lazău, Adina Berbecea

**Affiliations:** 1 Faculty of Industrial Chemistry and Environmental Engineering, Polytechnica University of Timişoara, Victoriei Square, No. 2, 300006 Timişoara, Romania; diana.daminescu@student.upt.ro (D.D.);; 2 Renewable Energy Research Institute-ICER, Polytechnica University of Timişoara, Gavril Musicescu Street, No. 138, 300774 Timişoara, Romania; nicoleta.nemes@upt.ro (N.S.N.);; 3 Soil Sciences Department, Banat’s University of Agricultural Sciences and Veterinary Medicine “King Mihai I of Romania” from Timisoara, Calea Aradului, No. 119, 300645 Timişoara, Romania; adinaberbecea@yahoo.com

**Keywords:** scandium, acid extraction, waste red mud, first-order kinetic model, second-order kinetic model

## Abstract

Industry represents a fundamental component of modern society, with the generation of massive amounts of industrial waste being the inevitable result of development activities in recent years. Red mud is an industrial waste generated during alumina production using the Bayer process of refining bauxite ore. It is a highly alkaline waste due to the incomplete removal of NaOH. There are several opinions in both the literature and legislation on the hazards of red mud. According to European and national legislation, this mud is not on the list of hazardous wastes; however, if the list of criteria are taken into account, it can be considered as hazardous. The complex processing of red mud is cost-effective because it contains elements such as iron, manganese, sodium, calcium, magnesium, zinc, strontium, lead, copper, cadmium, bismuth, barium and rare earths, especially scandium. Therefore, the selection of an extraction method depends on the form in which the element is present in solution. Extraction is one of the prospective separation and concentration methods. In this study, we evaluated the kinetic modelling of the solid–liquid acid extraction process of predominantly scandium as well as other elements present in red mud. Therefore, three acids (HCl, HNO_3_ and H_2_SO_4_) at different concentrations (10, 20 and 30%) were targeted for the extraction of Sc(III) from solid red mud. Specific parameters of the kinetics of the extraction process were studied, namely the solid:liquid ratio, initial acid concentration, contact time and temperature. The extraction kinetics of Sc(III) with acids was evaluated using first- and second-order kinetic models, involving kinetic parameters, rate constants, saturation concentration and activation energy. The second-order kinetic model was able to describe the mechanism of Sc(III) extraction from red mud. In addition, this study provides an overview on the mechanism of mass transfer involved in the acid extraction process of Sc(III), thereby enabling the design, optimization and control of large-scale processes for red mud recovery.

## 1. Introduction

Red mud is a waste material that results from the Bayer process of refining bauxite to produce alumina. The process was first discovered in 1897 by Carl Josef Bayer, and the procedure is generally carried out at high temperature and pressure to extract gibbsite (Al(OH)_3_) and/or boehmite (γ-AlOOH) from bauxite by treating its constituents in concentrated NaOH solution [1].

Red mud represents a material with higher environmental liability due to its strong alkalinity associated with incomplete NaOH removal [2]. Some other environmental problems are associated with the large volume of red mud due to higher demands for aluminium, lower particle size and its relatively complex chemical composition [3,4]. The chemical composition of red mud depends on several factors, but the main factor is the composition of the bauxite ore. The composition of bauxite also depends on the amount of red mud produced with between 0.33 and 2 tonnes of red mud generated per tonne of alumina produced [5,6].

The most significant environmental concern, apart from the massive amounts of red mud produced, is alkalinity. According to data reported in the literature, the main constituents are 70% red mud and 30% NaOH bauxite residue (pH = 11–13) [5].

In mineralogical terms, red mud is composed of metal oxides. Thus, the composition of red mud includes aluminium oxide (boehmite, diaspore), ferrous minerals (hematite, goethite and limonite), rutile, anatase, pyrite, calcite and dolomite. In addition to the above, new phases are formed during the Bayer process, i.e., sodalite and gibbsite [7].

Due to its high alkalinity and the fact that it is considered a hazardous waste, disposal methods, such as marine discharge, lagoons, dry stacking and dry cake disposal, are employed [5].

An important issue for all researchers is represented by the necessity to recover all valuable compounds from bauxite residue, but technological impediments associated with the low cost-effectiveness of the process limit the ability for upscaling [8]. Technologies developed to date allow the usage of red mud as an adsorbent, construction material, a pigment in the ceramics industry, or an absorbent for combustion gases [9]. On the other hand, red mud contains high-value components, especially rare metals, which represent a possible overall raw material given its chemical composition. These are some of the most important factors that are driving the research to find methods of recovering red mud [5].

Rare earth elements (REE) (scandium, yttrium, cerium, thulium, lanthanum, praseodymium, lutetium, dysprosium, etc.) have a wide range of applications and are used in new electronic devices, such as mobile phones, displays, high-capacity batteries, permanent magnets for wind energy, ceramics, etc. Scandium is used in the production of lenses and prisms used for movie or photographic equipment. In addition, scandium is also used in the field of astronomy [5,10,11,12].

Acid leaching of red mud using hydrochloric, sulphuric or nitric acid allows the recovery of scandium and other metals present. Thus, the aim of the present study was to recover scandium through acid leaching by establishing the kinetics of the extraction/leaching process. Mathematical modelling was considered an important step in the extraction process given that the influence of process parameters on the final outcome can be determined quickly, thereby minimising the number of experiments required. Solid–liquid extraction is a heterogeneous, multicomponent process in which the mechanism of mass transfer from solid to liquid occurs at varying rates. The application of the kinetic model and the parameter model analysis provides a good understanding of the extraction mechanism [13,14].

Solid–liquid extraction can be considered the opposite of adsorption (mass transfer occurs from a solid phase to a liquid phase, namely, the solvent). The kinetics of the extraction process appear to be similar to the kinetics of the adsorption process [15].

Therefore, the equation used for adsorption can be applied to extraction, and such kinetics are often described by a first-order kinetic reaction [15,16,17] and sometimes interpreted as a combination of two or three different mechanisms [18,19,20,21,22,23].

Moreover, the influence of other metal ions in the extraction/leaching process was also investigated to establish the competitive/selectivity of the method.

## 2. Materials and Methods

### 2.1. Composition of Red Mud

To determine the composition of red mud, an exact quantity of red mud was digested using an assisted microwave system. In this context, approximately 3 g of red mud was weighed to the nearest 0.001 g in a 250 mL reaction flask and moistened with approximately 0.5–1 mL of water. While stirring, 21 mL of concentrated hydrochloric acid was added followed by 7 mL of concentrated nitric acid, drop by drop if necessary, to the absorption flask. The refrigerant was added to the reaction flask. Then, allow the sample to sit for 16 h at room temperature to permit slow oxidation of the organic matter in the red mud. In this context, the reaction mixture temperature was slowly raised until the reflux conditions were reached and maintained for 2 h, ensuring that the condensation area is less than 1/3 of the height of the refrigerant. Then, the reaction was allowed to cool. Add the contents of the absorption bottle to the reaction flask through the refrigerant, rinsing both the absorption bottle and the refrigerant with an additional 10 mL of nitric acid. Allow the insoluble residue in the reaction flask to settle. Carefully pass the relatively free supernatant of the sediments obtained by decanting through a filter paper and collect the filtrate in a 100 mL volumetric flask. Pass all the initial extract from the reaction flask through the filter paper, then wash the insoluble residue on the filter paper with a minimum amount of nitric acid. Collect this filtrate with the first filtrate.

The metal ions content in the filtrate was measured by ICP-MS method using a PlasmaQuant 9100 ICPMS system (Analytic Jena, Jena, Germany).

### 2.2. Red Mud Characterization

#### Scanning Electron Microscopy (SEM) and Energy Dispersive X-ray Spectroscopy (EDX)

Morphological characterization of red mud was carried out by scanning electron microscopy (SEM) analysis. Chemical composition was revealed by energy dispersive X-ray spectroscopy (EDX) using a Quanta FEG 250 microscope (FEI, Hilsboro, OR, USA).

### 2.3. Fourier-Transform Infrared Spectroscopy (FT-IR)

Fourier-transform infrared spectroscopy was performed using the IRAffiniy-1S SHIMAZDU spectrophotometer (Shimadzu Corporation, Kyoto, Japan).

### 2.4. Red Mud Chemical and Granulometric Composition

The specific chemical composition of the red mud used was recorded in the XRD spectra, and the specific components were identified using a specific XRD database. Due to the importance of the particle size for the extraction process, the particle size distribution was determined using the Kacinski method [24].

### 2.5. Acid Leaching of Sc(III) Studies

In order to determine the mechanism of the Sc(III) extraction process and the metal ions present in the red mud, a series of parameters were studied, including the solid:liquid ratio, initial acid concentration, contact time and temperature.

### 2.6. Effect of Solid (Red Mud)/Acid Extraction Ratio (S:L Ratio)

To dissolve the oxides, a theoretical amount of acid of a certain concentration is used. To determine this amount of acid for 1 h, different amounts of red mud (0.25 g, 0.5 g, 0.75 g, 1 g and 2 g) were kept in contact with 25 mL acid HCl (OTIPURAN^®^ 37%, *w*/*w*), HNO_3_ (Silver Chemicals, 68%) and H_2_SO_4_ (Carl Roth, 96%) 20% (*w*/*w*) at 298 K. When the contact time was completed, the samples were filtered, and the Sc(III) concentration in the filtrate was determined using the ICP-MS technique.

### 2.7. Effect of Acid Concentration

Another important parameter in Sc(III) extraction is the concentration of the acid used for extraction. For this reason, extraction studies were carried out using three acids (HCl, HNO_3_ and H_2_SO_4_) at three different concentrations (10%, 20% and 30%, mass percentage). The S:L ratio chosen for the studies was 0.5 g red mud:25 mL acid. The contact time was 1 h, and the temperature was 298 K. When the contact time was completed, the samples were filtered, and the concentration of Sc(III) was analysed in The filtrate using ICP-MS.

### 2.8. Effect of Stripping and Temperature

In order to determine the influence of contact time and temperature on the efficiency of the extraction process, 0.5 g red mud was accurately weighed, and the three acids (HCl, HNO_3_ and H_2_SO_4_) 20% (*w*/*w*) were added. The samples were shaken at different times (30, 60, 90, 120, 180 and 240 min) in a thermostatic and stirred water bath and at different temperatures (298 K, 308 K and 318 K). The samples were shaken at 200 rpm.

When the contact time was completed, the samples were filtered, and the concentration of Sc(III) in the filtrate was analysed using ICP-MS.

### 2.9. Kinetic Models of Solid-Liquid Extraction: Activation Energy

The rate of extraction is proportional to a driving force, which is assumed to be given by the difference between *C_s_* and *C_t_*, where *C_s_* is the concentration of Me^n+^ soluble in the acidic medium [g/L] and *C_t_* is the concentration of Me^n+^ soluble in the acidic medium at time *t* [g/L]. The order of extraction and the rate constant of the extraction process are determined experimentally. There are several models that describe the extraction rate, such as a pseudo-one-order model, pseudo-two-order model, the Elovich model and the Roginsky-Zeldovich model.

Lagergren’s pseudo-first-order equation can be rewritten as a differential equation as follows [25,26]:

(1) $$\frac{{dC}_{t}}{d_{t}}=k_{1}(C_{s}-C_{t})$$

where *k*_1_ is the first-order extraction rate constant [1/min] and *t* is the time [min].

The linear form is provided:

(2) $${log(C}_{s}-C_{t})={log(C}_{s})-\frac{k_{1}}{2.303}t$$

From plotting (*C_s_* − *C_t_*) = *f*(*t*), the constant *k*_1_ is calculated from the slope of the line, and the extraction capacity at equilibrium is also evaluated for *C_s_* the concentration obtained at saturation.

The specific equation for the pseudo-second-order kinetic model can be described as follows [21,22,23]:

(3) $$\frac{{dC}_{t}}{d_{t}}=k_{2}(C_{s}-C_{t}{)}^{2}$$

or

(4) $$\frac{{dC}_{t}}{{\left(C_{s}-C_{t}\right)}^{2}}=k_{2}dt$$

where *k*_2_ is the pseudo-second-order extraction rate constant [L/g·min].

The linear form of Equation (3) is provided:

(5) $$\frac{t}{C_{t}}=\frac{1}{k_{2}C_{s}^{2}}+\frac{t}{C_{s}}$$

or:

(6) $$\frac{C_{t}}{t}=\frac{1}{{(1/k}_{2}C_{s}^{2})+(\frac{t}{C_{s}})}$$

The initial extraction rate, *h*, in $C_{t}/t$ where *t* is 0, can be described as follows: ${h=k}_{2}C_{s}^{2}$.

In this way, Equation (6) can be described as follows:

(7) $$\frac{t}{C_{t}}=\frac{t}{C_{s}}+\frac{1}{h}$$

From plotting $\frac{t}{C_{t}}=f(t)$, one can determine the initial extraction rate, *h*, the extraction capacity, *C_s_* and the pseudo-second-order rate constant, *k*_2_.

Also, we evaluate the activation energy of Sc(III) extraction using the Arrhenius equation and the speed constant from the pseudo-second-order model, which better describes the adsorption process.

$$\ln k_{2}=\ln A-\frac{E_{a}}{RT}$$

where *k*_2_ is the speed constant (g/min·mg), *A* is the Arrhenius constant (g·min/mg), *E_a_* is the activation energy (kJ/mol), *T* is the absolute temperature (K), and *R* is the universal gas constant (8.314 J/mol·K).

For red mud, it is possible to evaluate the activation energy using the mathematical equation associated with linear dependence as follows: ln *k*_2_ = f(1/T).

### 2.10. Error Analysis

To decide which model is suitable to describe the analysed experimental data, an error analysis must be performed. Such analysis is needed because the kinetic models used to describe extraction process contain different parameters, making proper model selection difficult. In this context, it is difficult to determine which model is the best one taking into account only the correlation coefficient (R2) [27]. The impact of different parameters on the fitting results can be eliminated using model selection criteria [28]. Different criteria were developed over time for the selection of the model that describes the studied process with maximum accuracy. One model is represented by the residual sum of squares (*RSS*) [27]:

$$RSS=\sum_{i}^{n}{\left(q_{i,exp}-q_{i,model}\right)}^{2}$$

In this context, the Akaike Information Criterion (*AIC*) represents most used criterion, but the accuracy of this criterion decreases when the ratio between the number of data points and the number of estimated parameters is less than 40 [28].

$${AIC}_{k}=Nln\frac{RSS}{N}+2P_{k}$$

where *N* is the number of data points, and *P_k_* is the number of the estimated parameters.

Model selection based on *AIC* is used to compute the *AIC* value for each model and further to rank them based on the *AIC* value, with the model with the lowest *AIC* considered as the best model [29]. This shortcoming of the *AIC* model has been eliminated by the development of the Bayesian Information Criterion (*BIC*) proposed by Schwarz and Akaike. The main reason for the development of this criterion was the need to have a selection method with different asymptotic properties compared with the *AIC*. The *BIC* name refers to Bayesian methods, but the mathematical formula it is similar to the *AIC* formula, differing only in the last term [29].

$${BIC}_{K}=Nln\frac{RSS}{N}+P_{k}lnN$$

where *N* is the number of data points, and *P_k_* is the number of the estimated parameters.

### 2.11. Extraction Process Selectivity

At the same time as the extraction of scandium from red mud by acid leaching, the metal ions present in the matrix composition are extracted according to the mineralogical structure. Thus, the content of sodium, magnesium, calcium, chromium, manganese, iron, zinc, strontium, lead, copper and cadmium is determined using ICP-MS. Lastly, a classification of the selectivity of the studied acids (HCl, HNO_3_ or H_2_SO_4_) was established.

## 3. Results and Discussions

### 3.1. Composition of Red Mud

In Table 1, the chemical composition of the solution obtained after red mud mineralization using aqua regia is presented.

It can be seen that red mud has ~100 mg/L Sc(III). This high Sc(III) content allowed further studies to be carried out in order to recover it using acids such as HCL, HNO_3_ or H_2_SO_4_.

### 3.2. Red Mud Characterization

**Figure 1:**
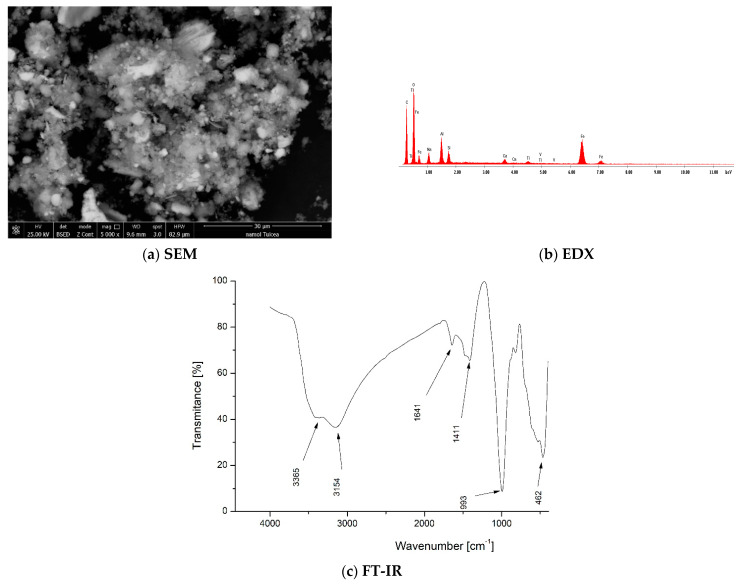
Red mud characterization using (**a**) scanning electronic microscopy (SEM); (**b**) energy-dispersive X-ray spectroscopy (EDX); and (**c**) Fourier-transform infrared spectroscopy (FT-IR).

The red mud was characterized by scanning electron microscopy (SEM) (Figure 1a), energy-dispersive X-ray spectroscopy (EDX) (Figure 1b), and Fourier-transform infrared spectroscopy (FT-IR) (Figure 1c). Obtained data are presented in Figure 1.

The SEM micrograph (Figure 1a) taken at 5000× magnification shows the powdery/grainy structure of red mud. Based on data depicted in Figure 1b, the elemental composition of red mud obtained from EDX spectrum supports the chemical composition shown in Table 1.

Figure 1c depicts the FT-IR spectra of red mud in which the presence of vibrations specific to the geomorphological compounds formed in red mud can be seen. Broad absorption bands at 3154 cm^−1^ and 3365 cm^−1^ are the vibrations specific to the Si-OH groups and –OH groups; these absorption bands are specific to the vibrations of the deformation bonds corresponding to water molecules [30]. At 1641 cm^−1^, there is Al-OH group-specific vibration, and the vibration bands due to H-O-H bending result from the formation of sodium silico aluminate hydrate or calcium silico aluminate hydrate [31,32].

The absorption band located at 1411 cm^−1^ may be associated with vibrations specific to calcium compounds, such as carbonates, and the absorption band at 993 cm^−1^ is associated with aluminosilicates [33] or the stretching vibration of the Si-O-Si group. The absorption band at 462 cm^−1^ appears to represent vibrations specific to Pb-O or Si-O-Si groups [34].

**Figure 2:**
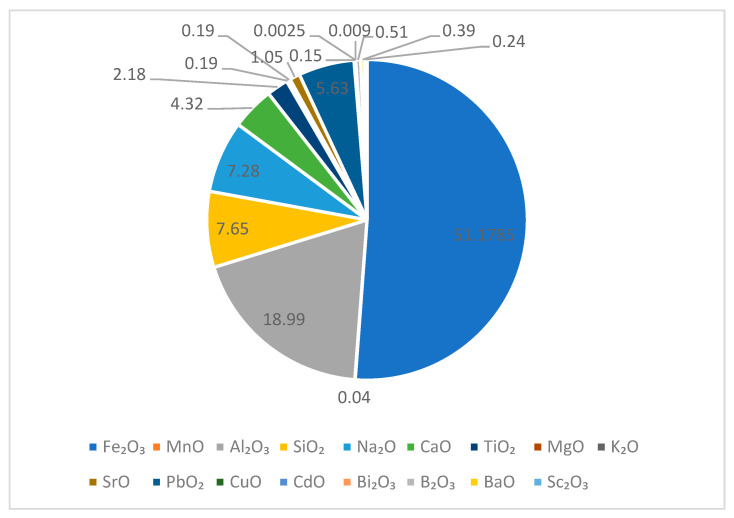
Red mud chemical constituents.

XRD analysis revealed the chemical composition of the red mud used during scandium recovery experiments. Consistent with the literature [35], the red mud consists of a mixture of different oxides incorporated into a based sodium salt solution, as can be observed in Figure 2.

The red mud structure and texture is in close correlation with the particle class sizes, depending on its mineralogical and chemical composition. In this context, the particle size distribution is an important parameter [35]. Particle size distribution was determined with the Kacinski method [24] dry red mud stored at room temperature.

**Figure 3:**
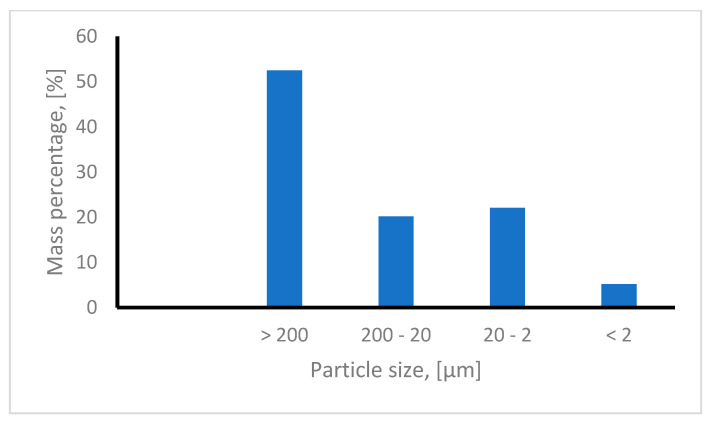
Particle size distribution.

From the data presented in Figure 3 can observe that the used red mud comprises four different particle classes:

- one particle fraction with a dimension greater than 200 μm accounting for 52.5% of the sample;
- one particle fraction with dimensions between 20 and 200 μm, accounting for 20.2% of the sample;
- a dust-like particle fraction with particle dimensions between 2 and 20 μm, accounting for 22.1% of the sample;
- one particle fraction consisting into very fine particles with dimensions less than 2 μm, accounting for 5.2% of the sample.

As previously mentioned, another important parameter is the red mud mineralogical composition.

### 3.3. Acid Leaching of Sc(III) Studies

In order to investigate the recovery of Sc(III) by extraction in acidic leach solution using three acids, HCl, HNO_3_ and H_2_SO_4_, the effects of the ratio of the solid/acid extraction (amount of solid/volume of acid extractant, *w*/*v*), acidic extractant concentration, contact time and temperature on the concentration of extracted Sc(III) were studied.

**Figure 4:**
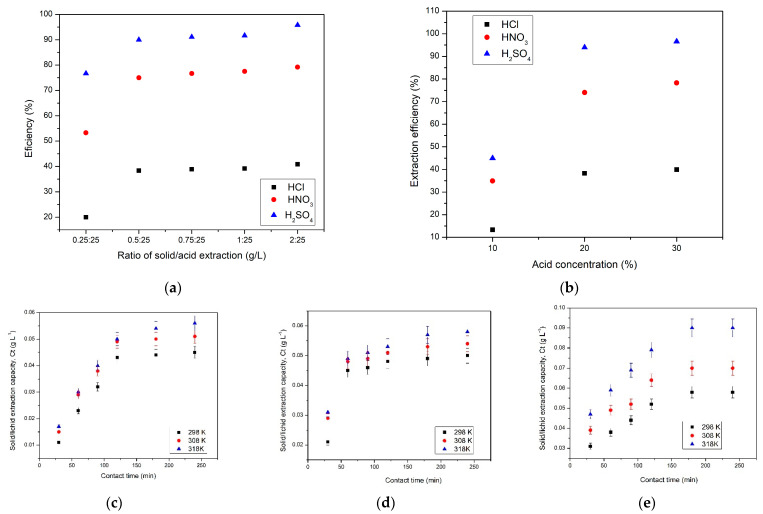
Acid leaching of Sc(III) studies. (**a**) Ratio of solid/acid extraction; (**b**) acid concentration; (**c**) contact time and temperature for 20% HCl; (**d**) contact time and temperature for 20% HNO_3_; and (**e**) contact time and temperature for 20% H_2_SO_4_.

Therefore, the effect of the ratio of solid/acid extraction (Figure 4a) and the effect of the concentration of the acid used for extraction (Figure 4b) on the efficiency of the extraction process are presented. In addition, the effect of contact time at 3 temperatures for the used acids (Figure 4c–e) on the acid extraction capacity is presented.

From the results presented in Figure 4a, we can observe that with an increasing solid/acid extraction ratio, the efficiency of the Sc(III) extraction process increases up to a certain point (ratio solid (red mud)/acid extraction 20% = 1/10), after which it remains approximately constant. If HCl is used as extractant, the efficiency increases but insignificantly to ~40% at a ratio solid (red mud)/acid solution = 1/10 HCl 20%. When using HNO_3_ as extractant, the efficiency increases to ~77.5% for the ratio solid (red mud)/acid extraction = 1/10 HNO_3_ 20%, and when using H_2_SO_4_ as extractant, the efficiency increases to ~91.5% for the ratio solid (red mud)/acid extraction = 1/10 H_2_SO_4_ 20%.

Figure 4b presents the effect of the acid concentration on the efficiency of the extraction process. Therefore, as the concentration of acid used for Sc(III) extraction from red mud increases, the efficiency of the process also increases. When using H_2_SO_4_ acid as an extractant, the process shows the highest efficiency of 96.6% compared to HCl (45%) or HNO_3_ (90%).

Figure 4c–e reveals that with increasing contact time, the extraction capacity of Sc(III) from red mud increases up to 120 min. After this contact time, the extraction capacity remains approximately constant. Also, temperature is another parameter that positively influences the extraction process of Sc(III) from red mud. When using HCl as an extractant, the extraction capacity is ~0.04 g/L at 298 K and 0.05 g/L at 318 K. When using HNO_3_ as an extractant, the extraction capacity is ~0.048 g/L at 298 K and 0.053 g/L at 318 K. When using H_2_SO_4_ as an extractant, the extraction capacity is ~0.052 g/L at 298 K and 0.08 g/L at 318 K. It is also evident from this study that sulphuric acid is the best extractant [36,37].

### 3.4. Kinetic Models of Solid–Liquid Extraction: Activation Energy

**Figure 5:**
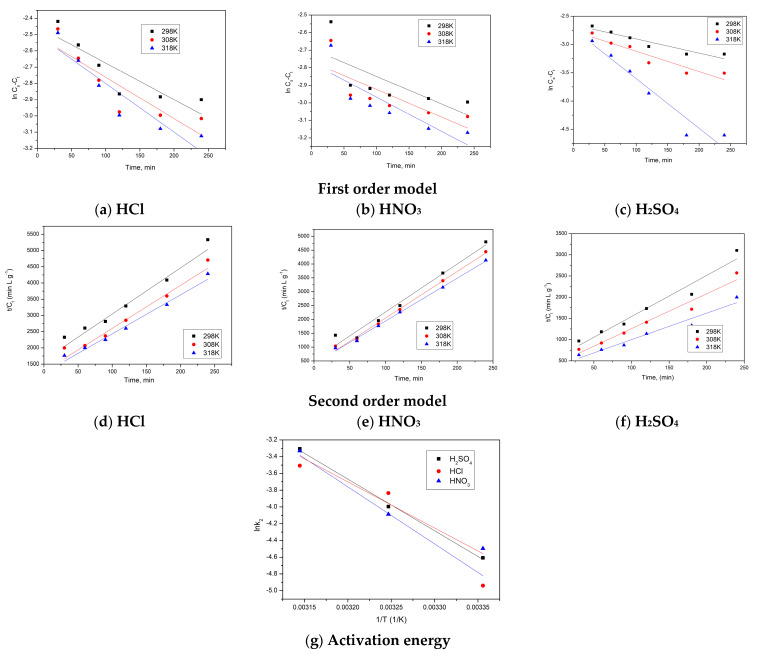
Kinetic models and activation energy.

In this study, first-order and second-order kinetic models were used to model the experimental data obtained for the extraction of Sc(III) from red mud with 3 acids, HCl, HNO_3_ and H_2_SO_4_. The activation energy was also determined using the Arrhenius equation. The obtained results are presented in Figure 5a–g.

From the experimental data, it can be seen that there is a gradual and slow increase in the efficiency of the extraction process until the maximum of extracted Sc(III) is reached. This phenomenon can be explained by Fick’s law. According to this law, at the beginning of the extraction process, the concentration gradient between the solid phase (red mud) and the liquid phase (acid) is high, and the diffusion is high. As the extraction process continues, the amount of Sc(III) extracted increases slowly until the maximum is reached [14,38].

For the pseudo-first-order kinetic model, the values obtained for k_1_, Cs, as well as the R^2^ coefficient, based on recorded experimental data are presented in Table 2 and Figure 5a–c. The value of the constant k_1_ increases with increasing temperature, with similar effects noted for all 3 acids studied as extractants. This finding correlates with the fact that temperature positively influences the extraction process. The R^2^ coefficient values vary between 0.898 and 0.937.

The pseudo-second-order model was also evaluated to study and describe the kinetics of the Sc(III) extraction process. The values of the kinetic constant k_2_, Cs, and the coefficient R^2^ obtained by modelling the experimental data for used extractants of Sc(III) from red mud as a function of temperature are presented in Table 2. The values of the coefficient R^2^ are close to 1, which allows us to state that this model better describes the kinetics of the extraction process [38]. The best kinetic model to describe the scandium extraction process was the Bayesian Information Criterion [27,28]. Obtained values of the BIC are depicted in Table 2. According to Cao et al. [28] and Lima et al. [39], the kinetic model which best describe the studied extraction process it is the model presenting the most negative BIC value. In this context, from the data presented in Table 2, one can conclude that the pseudo-second-order model better describes the Sc(III) extraction process.

Table 3 shows the activation energy values, which are similar regardless of the nature of the acid used as an extractant, with values of 5.2–5.5 Kj/mol.

### 3.5. Extraction Process Selectivity

Because sulphuric acid is the acid that extracts Sc(III) with the best efficiency, the optimal conditions of Sc(III) extraction are obtained, namely: ratio solid (red mud)/H_2_SO_4_ 20% = 1/10, contact time 120 min, and temperature 298 K. The content of metal ions extracted simultaneously with Sc(III) was determined by ICP-MS, and data are presented in Table 4. The extraction order is as follows: Sc > Na > Ca > Zn > Mg > Cd > Cu > Cr > Mn > Pb. Over 96.6% Sc(III) is extracted, and 0.02% Pb(II) is extracted.

## 4. Conclusions

Scandium can be extracted from red mud with acids at room temperature. The process is influenced by the solid (red mud)/acid extractant ratio, the acid concentration, the nature of the acid and the contact time. Thus, the optimal conditions are noted as follows: ratio solid (red mud)/acid extraction = 1/10, sulphuric acid concentration 20%, and contact time 120 min. Under these conditions, the extraction efficiency of Sc(III) is ~96.6%. Taking into account the data obtained by modelling the Sc(III) extraction process and correlating them with the BIC calculations can conclude that this process is better described by the pseudo-second-order kinetic model.

It can be stated that sulphuric acid shows selectivity for Sc(III) extraction with the best efficiency, taking into account that under the optimal conditions the established extraction order is as follows: Sc > Na > Ca > Zn > Mg > Cd > Cu > Cr> Mn > Pb.

Based on the kinetic studies carried out, it was established that the second-order model best describes the extraction process and that E_a_ is 5.5 kJ/mol.

The information presented in this study can be improved by designing and optimizing future extraction processes to reduce costs.

## Figures and Tables

**Table 1:** Chemical composition of the solution obtained after red mud after mineralization (wt, %).

Na	Mg	Ca	Cr	Mn	Fe	Zn	Sr	Pb	Cu	Cd	Bi	B	Ba	Sc	Other
5.41 ± 0.27	3.82 ± 0.19	12.7 ± 0.64	0.06 ± 0.003	3.18 ± 0.16	63.7 ± 3.18	0.05 ± 0.0025	1.18 ± 0.06	7.01 ± 0.35	0.18 ± 0.009	0.03 ± 0.0015	0.05 ± 0.0025	0.67 ± 0.033	0.64 ± 0.032	0.32 ± 0.016	0.97 ± 0.048

**Table 2:** Kinetic parameters and activation energy.

**Pseudo First-Order Model**						
**Acid Nature**	**Temperature, K**	***k*** **_1_** **,** **min^−1^ (×10^−3^)**	**Saturation Concentration,** **C_s_, g L^−1^**	**R^2^**	**RSS**	**BIC**
H_2_SO_4_	298	2.5	0.063	0.898	4.000	−2.773
	308	3.6	0.065	0.8908	9.000	−4.395
	318	8.8	0.070	0.9379	16.000	−5.545
HCl	298	2.2	0.059	0.8097	4.000	−2.773
	308	3.4	0.069	0.891	6.000	−3.665
	318	8.5	0.080	0.9382	7.000	−3.973
HNO_3_	298	1.5	0.065	0.896	4.000	−2.773
	308	3.4	0.069	0.8909	6.000	−3.665
	318	8.7	0.077	0.9376	32.000	6.962
**Pseudo Second-Order Model**						
**Acid Nature**	**Temperature, K**	***k*** **_2_** **g L^−1^∙min^−1^ (×10^−3^)**	**Saturation Concentration,** **C_s_, g L^−1^**	**R^2^**	**RSS**	**BIC**
H_2_SO_4_	298	9.96	0.07	0.9917	400.00	−11.983
	308	18.40	0.08	0.9919	625.00	−12.875
	318	36.64	0.11	0.9917	3025.0	−16.029
HCl	298	7.15	0.06	0.9918	100.00	−9.210
	308	21.60	0.09	0.9921	625.00	−12.875
	318	29.95	0.10	0.9918	900.00	13.605
HNO_3_	298	11.15	0.07	0.9917	400.00	−11.983
	308	16.78	0.08	0.9923	225.00	−10.832
	318	35.79	0.11	0.9921	1600.0	−14.755

**Table 3:** Calculated activation energy.

Activation Energy		
Acid Nature	Ea, KJ/mol	R^2^
H_2_SO_4_	5.50	0.9973
HCl	5.25	0.9982
HNO_3_	5.43	0.9993

**Table 4:** Extraction process selectivity.

Ion	Na	Mg	Ca	Cr	Mn	Fe	Zn	Sr	Pb	Cu	Cd
Extraction efficiency (%)	93.67	7.50	17.29	1.22	0.08	0.21	9.97	0.15	0.02	1.57	3.95
